# Barriers to diabetes medication adherence in North West Ethiopia

**DOI:** 10.1186/2193-1801-3-195

**Published:** 2014-04-17

**Authors:** Solomon Mekonnen Abebe, Yemane Berhane, Alemayehu Worku

**Affiliations:** Department of Physiotherapy, College of Medicine and Health Sciences, University of Gondar, Gondar, Ethiopia; Addis Continental Institute of Public Health, Addis Ababa, Ethiopia; School of Public Health, Addis Ababa University, Addis Ababa, Ethiopia

**Keywords:** Low-adherence, HbA_1c_, Poor glycemic control, Traditional medicine

## Abstract

**Background:**

Low adherence to prescribed diabetes medications is one of the major reasons to poor glycemic control in developing countries. Therefore, this study attempted to assess the magnitude of medication adherence and factors associated with it among adult persons with diabetes in northwest Ethiopia.

**Method:**

This study utilized a cross sectional study design with internal comparison. The study population was adult persons with diabetes attending the Diabetes Referral Clinic of Gondar University Hospital. Adherence was assessed using the eight-item Morisky Medication Adherence Scale (MMAS-8). In addition laboratory tests and chart reviews were carried out to collect relevant data. Ordinary logistic regression was used to identify factors associated with adherence.

**Result:**

A total of 391 patients were studied. Based on the MMAS-8 scale, the self-reported adherence to diabetic medication was low for 25.4% [95% CI: 21, 29] of the patients, medium for 28.7% [95% CI: 24, 33], and high for 45.9% [95% CI: 41, 50] of the patients. The Mean (±SD) of glycosylated hemoglobin for the low adherence group was 8.2% (±2.1). It was 8.1% (±2.0), for the medium, and 7.4% (±1.6) for the high adherence group. In the multivariate analysis poor wealth status (AOR = 1.99; 1.15, 3.43), using traditional treatment (AOR = 2.90; 1.03, 8.15), and service dissatisfaction (AOR = 2.23; 1.04, 4.80) were significantly associated with low adherence to prescribed diabetic medications.

**Conclusion:**

Over half of the persons with diabetes did not adhere to medications. Adherence was poor among users of traditional treatment and those dissatisfied with services. Developing a more intensive communication strategies and improving the quality of services could improve the level of adherence.

## Background

Poor treatment adherence that contributes to suboptimal glycemic control continues to be one of the major barriers to effective diabetes management. The World Health Organization has reported that as many as fifty percent of the patients with chronic diseases do not take their medications as prescribed (Brown and Bussell 2011; Reynolds et al. 2004). Effective and successful glucose control requires appropriate and timely use of medication over the entire period of treatment, which is often lifelong. Different studies have shown that adherence to diabetes treatment has been highly varied and may range from 1.4 to 88.0% (Kalyango et al. 2008; Raum et al. 2012; Sankar et al. 2013).

Low adherence to prescribed diabetes medications accounts for 30% to 50% of treatment failures, leading to worse treatment outcomes and which cause damages to vital organs (Medicine ARftACoP 2011). Treatment failure is in turn associated with reduced treatment benefits and can have a negative financial burden on both individual patients and the society at large (Wroth and Pathman 2006; DiMatteo et al. 2002). In fact, effective diabetes management often needs coordinated quality services (Tricco et al. 2012).

Previous studies showed that therapy with multiple drugs, poor appointment keeping, poor patient-provider communication, and low patient education were factors which increased poor medication adherence. In addition, medication adherence has also been influenced by local culture and religious affiliations that influence on individual medication behaviors (Peyrot and Rubin 1994; Ciechanowski et al. 2001; Weinger et al. 2011; Rhee et al. 2005; Balkrishnan et al. 2003; Collins-McNeil et al. 2012). Despite the fact that giving due recognition to diabetes mellitus as one of the major health issues of the middle age and the elderly necessitates the gathering of relevant information on medication behaviors and factors associated thereof, studies on patient medication adherence are very limited in Ethiopia (Collins-McNeil et al. 2012; Ho et al. 2009; Wabe et al. 2011; Abebe et al. 2013). Such information is very helpful for improving access and the quality of services. Thus, the objective of the study was to determine the magnitude of medication adherence and associated factors among adult persons with diabetes.

## Methods

This study utilized a cross-sectional hospital based design and was conducted at the Diabetic Clinic of the University of Gondar Referral Hospital. Opened two decades ago, the clinic was serving over 8,000 persons with diabetes at the time of the study.

The study population comprised confirmed persons with diabetes aged 18 years and older attending the Diabetes Referral Clinic. Individuals with a follow-up duration of less than twelve months were excluded in order to avoid the immediate period following diagnosis and to obtain adequate time for observing adherence (Donnan et al. 2002). Sample size was determined using two proportion formula considering a 95% confidence level, power of 80%, and various factors that were known to influence diabetes medication adherence, and resulted in a sample size of 407 (Sabanayagam et al. 2009; Khattab et al. 2010). The follow-up record book was used as a sampling frame to randomly select eligible study subjects. Study participants were selected using a systematic sampling procedure; every second person aged 18 years and older who visited the Clinic in the months of January and February 2013 was selected into the study on a daily basis.

### Data collection

Data were collected by interviewing eligible subjects using a pretested structured questionnaire. Data collectors were trained laboratory technicians and nurses. The questionnaire, which was administered in the local language (Amharic), included questions that assessed diabetic risk factors, demographic characteristics, wealth status, duration of diabetes, lifestyle, clinic attendance, and drug regimen. We also gathered information on self-perception by asking how satisfied they were with the care they received at the current diabetic clinic (we rated the responses on a three level scale as very dissatisfied, satisfied and very satisfied). In addition, physical measurements were taken using standardized techniques and calibrated equipment. Height was measured using a stadiometer; participants stood in erect posture without shoes. Body mass index (BMI) was calculated as the ratio of weight in kilograms to the square of height in meters. Waist girth was measured by placing a plastic tape to the nearest 0.5 cm horizontally, midway between the 12th rib and iliac crest on the mid-axillary line. Hip circumference was measured around the widest portion of the buttocks (Grundy et al. 2004).

Adherence was assessed using the eight-item Morisky medication adherence scale (which asked, Do you sometimes forget to take your pills? People sometimes miss their medications for reasons other than forgetting? Thinking over the past two weeks, were there any days when you did not take your medicine? Have you ever cut back or stopped taking your medication without telling your doctor because you felt worse when you took it? When you travel or leave home, do you sometimes forget to bring along your medication? Did you take your medicine yesterday? When you feel like your DM is under control, do you sometimes stop taking your medicine? And taking medication everyday is a real inconvenience for some people; do you ever feel hassled about sticking to your treatment plan?) (Peyrot and Rubin 1994). Biochemical tests (HbA1c tests) were done for each study participant.

### Data management

Six laboratory technicians, four nurses, and two supervisors were trained by the principal investigator to assist during data collection. Biochemical tests (HbA1c) were carried out using 902 Automatic Analyzer with Roche/Hitachi kit following a minimum of 10 hours fasting period. To ensure the quality of the interview and the acquisition of quality data, random checks were carried out by field supervisors and the principal investigator. Data entry, cleaning, and coding were done using Epi-info version 3.5.3.

### Statistical analysis

Adherence was assessed using the eight-item Morisky Medication Adherence Scale (MMAS-8). Accordingly, individuals were classified as low-adherence if the mean score was less than 6, medium adherence if the mean score was 6 and 7, and high adherence if the mean score was 8 (Raum et al. 2012; Ho et al. 2009; Al-Qazaz et al. 2010). Glycemic control level was calculated using a Cut-point HbA1c 7%. Thus, those who scored 7% or more were coded as having poor glycemic control, and those with HbA1c score less than 7% were coded as having good glycemic control (Shalansky et al. 2004; Moreira et al. 2010). Both extremes, poor and rich wealth status, were compared to the reference (medium wealth status) since they are both risk for poor glycemic control.

Poor glycemic control proportions were computed for both Type 1 and Type 2 persons with diabetes. Ordinary logistic regression was applied to test the presence of association with three level outcome; low-adherence, medium adherence and high Adherence. The results were considered statistically significant at P ≤ 0.05. The independent variables were selected based on prior evidences in the literature and their effect in the current analysis; variables with the P-value of <0.2 in the bivariate analysis were selected for the model to ensure marginal confounders are considered in the final analysis.

### Ethical statement

The study protocol was approved by the IRB of the University of Gondar. Patients were recruited voluntarily and informed written consent was obtained from each study participant. For the sake of privacy and confidentiality no personal identifiers, such as name, were collected.

## Results

A sample of 407 persons with diabetes joined this study; sixteen of them refused to participate yielding a response rate of 96.01% (391 out of 407). Of these, 280 (71.6%) were Type 2 diabetics. The Mean age (±SD) of the study group was 50.4 (±15.2) years; 35.8 (±13.4) were Type 1 persons with diabetes, and 56.1 (±11.5) years Type 2 persons with diabetes. The mean duration since diagnosis with diabetes was 6.8 (±5.1) years.

**Figure 1:**
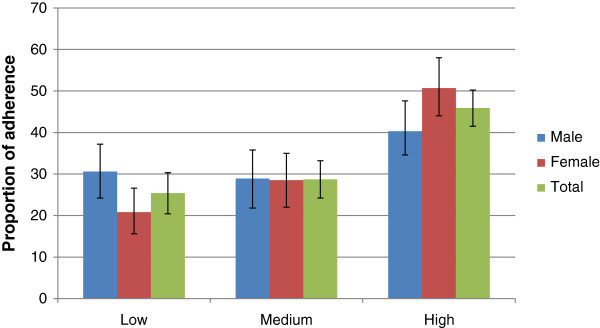
**Proportion of adherence by sex with 95% CI error bar among adults age 18 and above years person with diabetes, North West Ethiopia, 2013.**

Self-reported adherence to medication measure by MMAS-8 scale was low for 25.4% [95% CI: 21, 29], medium for 28.7% [95% CI: 24, 33], and high for 45.9% [95% CI: 41, 50] of the study subjects (Figure 1).

Overall, about a third, 138 (35.4%), of the study subjects had good glycemic control (HbA1c < 7%). Glycemic control was better achieved with improved medication adherence, and those who reported high medication adherence were more likely to have good glycemic control (Table 1).

**Table 1:** **The relationship between adherence categories and glycemic control groups: the Morisky scale adherence categories and glycemic control groups in Gondar University Referral Hospital (2013) Ethiopia**

Glycemic level	Low adherence (score < 75%)	Medium adherence (score ≥ 75 < 100)	High adherence (score = 100)	P-value
Good control (HbA1c < 7% )	28 (20.3%)	29 (21.0%)	81 (58.7%)	*P = 0.001*
Poor control (HbA1c ≥ 7%)	71 (28.2%)	83 (32.9%)	98 (38.9%)	
Total	99 (25.4%)	112 (28.7%)	179 (45.9%)	

A significant relationship between medication adherence and HbA1C categories (x^2^ = 14.22) was found.

The multivariate ordinal logistic regression analysis showed that people with poor wealth status (AOR = 1.99; 95% CI: 1.15, 3.43), those dissatisfied with clinic services (AOR = 2.23: 95% CI: 1.04, 4.80), patients receiving noninsulin regimen (AOR = 2.31; 95% CI: 1.50, 3.47) and patients consulting traditional healers (AOR = 2.90; 95% CI: 1.03, 8.15) were more likely to adhere poorly to diabetes medications (Table 2).

**Table 2:** **Multivariate ordinary logistic regression analysis of factors associated with low-adherence among person with diabetes in Gondar Referral Hospital of Gondar, North West Ethiopia (2013)**

Variable	n	Low to medium medication adherence n (%)	Adjusted OR [95% CI]
**Age in year**			
≤ 24	29	20 (68.9)	1.00
25–44	98	61 (62.2)	1.12 [0.51,2.46]
45–64	190	96 (50.5)	0.56 [0.25,1.24]
≥65	73	34 (46.6)	0.47 [0.19,1.14]
**Sex**			
Female	207	102 (48.3)	0.73 [0.47,1.13]
Male	184	109 (59.6)	1.00
**Wealth Index**			
Medium	118	55 (46.6)	1.00
Poor	135	79 (58.9)	1.99 [1.15,3.43]
Rich	138	77 (55.8)	1.56 [0.94,2.59]
**Insulin treatment**			
No	192	115 (60.2)	2.31 [1.50,3.47]
Yes	199	96 (48.2)	1.00
**Level of Education**			
Illiterate	143	69 (48.2)	1.00
Grad 1–6	50	25 (50.0)	1.04 [0.54,2.02]
Grad 7–12	120	65 (54.2)	1.29 [0.76,2.05]
Diploma and above	78	52 (67.5)	1.79 [0.94,3.43]
**Transport**			
Waking	90	55 (61.1)	0.72 [0.45,1.14]
Bus	300	156 (52.0)	1.00
**Reported using traditional medication**			
No	375	200 (53.3)	1.00
Yes	15	11 (73.3)	2.90 [1.03,8.15]
**Service satisfaction level**			
Dissatisfied	36	21 (58.3)	2.23 [1.04,4.80]
Satisfaction	212	124 (58.5)	1.53 [1.03,2.34]
Highly satisfied	141	66 (46.8)	1.00
**Health education**			
**No**	43	19 (44.2)	1.00
**Yes**	347	192 (55.3)	1.89 [0.97,3.70]

## Discussion

This study uncovered that only about a third of the persons with diabetes achieved a good glycemic control. Patients who were more likely to demonstrate poor adherence to prescribed diabetes medication were users of traditional medication, receivers of noninsulin treatment, persons with poor wealth status, and those dissatisfied with services.

The proportion of low-adherence seen was comparable with that of several other studies conducted in Sub-Saharan Africa (Kalyango et al. 2008; Al-Qazaz et al. 2010; Fedrick and Justin-Temu 2012; Teklay et al. 2013). However, lack of standard measurements and use of different definitions make comparison challenging. It is important for health care providers to consider low medication adherence as a factor contributing to poor glycemic control (Lau and Nau 2004). Thus, designing strategies to improve medication adherence might improve glycemic control and there-by decrease the rate of chronic complications related to diabetes.

This study showed a significant association between poor socio-economic status and low-adherence. These findings are in agreement with other studies that showed food insecurity and poor socioeconomic status were associated with low-adherence and poor glycemic control in persons with diabetes (Raum et al. 2012; Wabe et al. 2011; Larranaga et al. 2005; Seligman and Schillinger 2010; Seligman et al. 2012). Individuals with low socioeconomic status cannot access education, information, transportation and obtain the required drugs on time. This may increase patients’ difficulty to follow diabetes treatment (Seligman et al. 2012; Drewnowski and Darmon 2005). If the patient feels that the cost of therapy is a financial burden, the compliance with therapy will definitely be threatened causing low adherence (Jin et al. 2008).

An additional reason for patients’ low-adherence to diabetes treatment was their belief in traditional healers and preference to use such treatment (Haque et al. 2005). Integrating traditional and spiritual practices with modern health care system is an area for further research as the disease requires life-long care, perhaps from both systems in developing countries (Mann et al. 2009). Chronic diseases and their treatment is poorly understood within traditional and faith based healing systems, and may thus affect medication adherence (de-Graft Aikins et al. 2010). Poor adherence can also be an active process whereby patients choose to deviate from the treatment regimen through a rational decision process in which they weigh the risks and benefits of treatment against any adverse effects (Raum et al. 2012; Rutebemberwa et al. 2013).

Failing to elicit the history of the use of alternative, traditional, or supplemental therapy from patients due to ineffective communication between the patient and the health care provider would be a missed opportunity to improve medication adherence (Brown and Bussell 2011). A doctor-patient relationship based on trust is important to encourage patients to tell the health care provider about alternative medicine they are using honestly (Haque et al. 2005).

Dissatisfaction with the service received at the diabetic clinic was significantly associated with low adherence. Several reports have shown that service satisfaction is an important factor for good adherence among persons with diabetes (Biderman et al. 2009; Kocarnik et al. 2012). Patients dissatisfied with services provided at clinics might not be willing to attend the follow-up clinic. So dissatisfaction could be a barrier to achieving high adherence level and good glycemic control.

Our study showed that patients receiving noninsulin treatment were more likely to adhere with treatment compared to those receiving insulin treatments. This finding is consistent with those of other studies that showed lower adherence to oral medication agents among Type 2 persons with diabetes (Brown and Bussell 2011; Wabe et al. 2011; Lin et al. 2004; Farsaei et al. 2011). Patients receiving insulin treatment are more likely to take the disease more seriously, and are more likely to be symptomatic while non-insulin treated Type 2 persons with diabetes are usually asymptomatic (Tiv et al. 2012).

One of the limitations of this study was that self-reported medication adherence were used as a measure of the level of adherence. Since the study was conducted at a clinic setting, a social desirability bias might have creeped in. However, during the orientation efforts were made to explain about the aim of the study, the confidentiality of the research process and its zero effect on the services they are receiving. The Morisky medication adherence scale has been a validated and one of the most widely used self-reported measures of adherence. Thus, it is unlikely that our estimates are seriously underestimated.

## Conclusion

Low-adherence to medication regimens is common among persons with diabetes and is substantially related to poor glycemic control. Use of traditional medication and dissatisfaction with services were important factors associated with low adherence. Since the above risks are modifiable factors, addressing these risks through improving service availability, enhancing family support, providing more intensive communicative strategies and better health education could improve the level of adherence.
